# Precession-band variance missing from East Asian monsoon runoff

**DOI:** 10.1038/s41467-018-05814-0

**Published:** 2018-08-22

**Authors:** S. C. Clemens, A. Holbourn, Y. Kubota, K. E. Lee, Z. Liu, G. Chen, A. Nelson, B. Fox-Kemper

**Affiliations:** 1 Earth, Environmental, and Planetary Sciences, Brown University, Providence, 02912 RI USA; 2 Institute of Geosciences, Christian-Albrechts-University, 24118 Kiel, Germany; 3 Geology and Paleontology, National Museum of Nature and Science, Tsukuba, 110-8718 Japan; 4 Ocean Science, Korea Maritime and Ocean University, Busan, 49112 South Korea; 5 Atmospheric Sciences Program, Department of Geography, The Ohio State University, Columbus, 43210 OH USA; 6 Institute of Earth Environment, Chinese Academy of Sciences, 710061 Xi’an, China

## Abstract

Speleothem CaCO_3_ δ^18^O is a commonly employed paleomonsoon proxy. However, inferring local rainfall amount from speleothem δ^18^O can be complicated due to changing source water δ^18^O, temperature effects, and rainout over the moisture transport path. These complications are addressed using δ^18^O of planktonic foraminiferal CaCO_3_, offshore from the Yangtze River Valley (YRV). The advantage is that the effects of global seawater δ^18^O and local temperature changes can be quantitatively removed, yielding a record of local seawater δ^18^O, a proxy that responds primarily to dilution by local precipitation and runoff. Whereas YRV speleothem δ^18^O is dominated by precession-band (23 ky) cyclicity, local seawater δ^18^O is dominated by eccentricity (100 ky) and obliquity (41 ky) cycles, with almost no precession-scale variance. These results, consistent with records outside the YRV, suggest that East Asian monsoon rainfall is more sensitive to greenhouse gas and high-latitude ice sheet forcing than to direct insolation forcing.

## Abstract (web summary)

The underlying mechanisms driving the variability of the East Asia Monsoon during the late Pleistocene remain unclear. Here, the authors present a record of local precipitation and runoff from the East Chain Sea, which indicates strong sensitivity to greenhouse gases and high latitude ice sheet forcing.

## Introduction

The study of orbital-scale climate change during the late Pleistocene is unique in that the signal-to-noise ratio is large, the external forcing (insolation) is known, and the critical internal forcings (greenhouse gases and terrestrial ice volume) are extremely well constrained. As such, it should be possible to assess the underlying mechanisms driving climate change at this time scale, as well as the relative sensitivity to these forcings. The orbital time scale (10^4^ to 10^5^ yr) is also important because the frequency and amplitude of abrupt, centennial, and millennial-scale events may vary with changes in the longer-term mean state^1,2^. Here the focus is on orbital-scale variability in the East Asian Monsoon (EAM), defined as variance concentrated at periods (period = 1/frequency) associated with the eccentricity (~100 ky), obliquity (~41 ky), and precession (~23 and ~19 ky) characteristics of the Earth–Sun orbital geometry^3,4^. Understanding changes in monsoon precipitation as function of changing insolation, global ice-volume, and greenhouse gases is a primary goal of paleoclimate research given the prospect of climate-induced changes in summer-season rainfall impacting agriculture, and food production.

An extensive array of paleomonsoon proxies has been developed and applied throughout the Indian Monsoon and EAM regions in an effort to reconstruct and understand the mechanisms driving changes in rainfall as recorded in loess, lake, cave, and marine archives^5–9^. Most EAM proxy records contain variance associated with eccentricity, obliquity, precession, and heterodynes thereof; usually with the largest concentrations of variance at eccentricity, obliquity, or orbital-associated heterodynes^10–12^. This indicates that the EAM responds strongly to global ice volume and greenhouse gasses^9–12^ (also dominated by eccentricity-band and obliquity-band variance), as well as insolation forcing (dominated by precession-band variance). Heterodyne variance in EAM and other records^10^ arises from incorporation of variance from climatic processes operating at different primary orbital periods^13^ and can be calculated by adding and subtracting primary orbital frequencies (frequency = 1/period). For example, the interaction of variance at eccentricity periods of 130.6, 123.8, 98.7, and 94.7 ky with obliquity at 41.1 ky yields heterodyne spectral peaks at periods ranging from 28.7 to 31.3 ky and from 60 to 72.6 ky (e.g., 1**/**98.7 ± 1**/**41.1 = 1**/**29 and 1/70.4).

A primary exception is the composite speleothem δ^18^O record from the Yangtze River Valley (YRV), characterized by a spectrum almost exclusively dominated by precession-band variance^14,15^. This unique, exceptionally well-dated record and associated spectrum have been interpreted as reflecting East Asian summer-monsoon rainfall, varying dominantly and directly in response to changes in northern hemisphere summer insolation^14–16^, with little influence from global ice volume and greenhouse gasses. A great deal of effort has been spent testing this hypothesis, with a particular focus on the extent to which the YRV speleothem δ^18^O record (δ^18^O_cave_) should be interpreted as East Asian rainfall amount^17–19^, upstream changes in evaporative source regions, moisture transport paths, and evaporation and condensation processes along the transport path^8,9,20–26^, or changes in seasonality and frontal position^27–29^. Beyond large-scale ocean and atmospheric processes, cave ventilation seasonality^30^ and soil zone and epikarst dynamics^31,32^ have been discussed as well, including threshold processes associated with abrupt changes in soil evaporation and water flow through the soil horizon^25,33^.

Here four new highly-resolved records from International Ocean Discovery Program (IODP) Site U1429 in the East China Sea (ECS) are presented and used to reconstruct local seawater δ^18^O (δ^18^O_sw_), a parameter known to vary linearly with salinity; in this case varying as a function of YRV runoff and direct precipitation to the ECS. The ECS planktonic foraminifer δ^18^O record replicates, to an extraordinary degree, the precession- and sub-orbital-scale variance found in onshore δ^18^O_cave_. However, when corrected for the effects of changing local temperature and global seawater δ^18^O, the resulting local δ^18^O_sw_ record lacks significant precession-band variance, in contrast to the precession-dominated YRV δ^18^O_cave_ spectrum. Instead, local δ^18^O_sw_ is dominated by eccentricity- and obliquity-band variance as well as two heterodynes predicted by interaction of processes operating at these bands. The δ^18^O_sw_ spectrum is consistent with records across the EAM region^9–12,34^ that are also dominated by 100-, 41-, and heterodyne-derived variance, indicating that EAM variability is not driven dominantly and directly by insolation forcing, but is sensitive to internal forcing associated with changes in global-scale ice volume and greenhouse gas forcing^10,11,34^.

## Results

### Oceanography and climatology

**Fig. 1:**
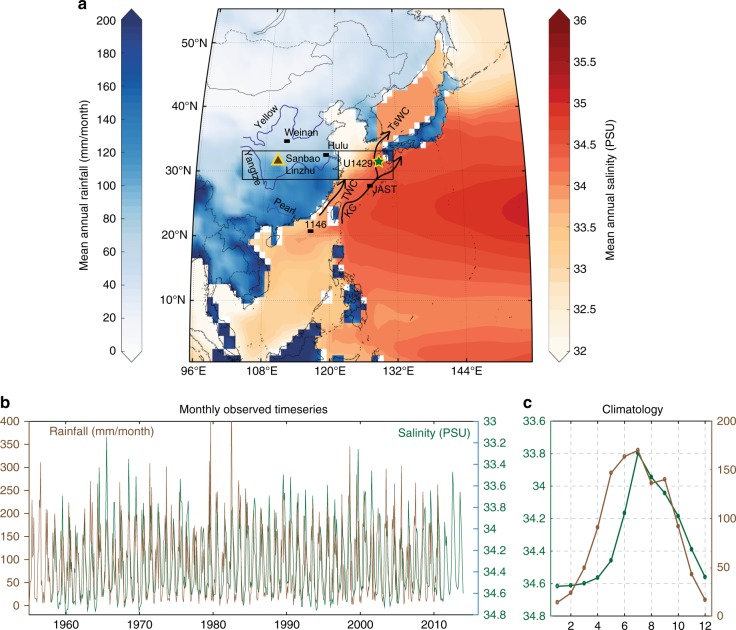
Modern precipitation and salinity climatologies, rivers, and surface currents. **a** Locations of IODP Site U1429 and sediment trap site JAST-01^48^ in the East China Sea. δ^2^H_wax_ records precipitation isotopes from the Pearl River Valley^34^ and the Loess Plateau^10^, deposited at IODP Site 1146 and Weinan, respectively. Boxes are area averages for CCSM3 model results. Kuroshio current (KC) and Tsushima warm current (TsWC) split south of U1429^90, 91^. Taiwan warm current (TWC) enters from the south. Sanbao, Linzhu, and Hulu caves are the locations from which the composite δ^18^O_cave_ record was constructed^15^. **b** Modern rainfall in the Yangtze River Valley (brown) is highly correlated to salinity at East China Sea Site U1429 (green). At the annual cycle with the lag removed, *r* = 0.89 and the result is significant at *p* < 0.01. **c** Monthly climatology from **b**. Salinity from ORAS4^92^. Rainfall from GPCC^93^. Base-map created using commercially available MATLAB software

Site U1429 (31.62°N, 129°E, 732 mbsl)^35^ is located at the north end of the Okinawa Trough (Fig. 1). Winters are characterized by cool sea surface temperature (SST) and strong winds (~18 °C, 5–7  ms^−1^) while summers are characterized by warm SST and weak winds (~28 °C, 1–2  ms^−1^)^36^. The surface moisture balance (precipitation minus evaporation; P − E) is positive in the summer and negative in the winter, consistent with seasonal differences in insolation, winds, and precipitation. Surface water salinity is also seasonal, due to increased P − E and YRV summer runoff. Modern salinity at Site U1429 ranges from 33.5 in the summer to 34.7 in the winter^37^ and is very highly correlated with rainfall over the YRV (Fig. 1). This strong correlation extends to the interannual scale as well; YRV flood and drought conditions related to El Niño and La Niña variability can alter salinity by +1 and −6 psu (respectively) out to 500 km offshore^38^ and are accompanied by interannual variability in chororphyll-*a*^39^. These observations are consistent with strong correlation (*r* > 0.97) of seawater δ^18^O with chlorinity and salinity, which indicate near-complete mixing of Yangtze River outflow and ocean waters in the estuary^40^ and in the eastern ECS^41^.

The strong link between modern YRV rainfall and offshore salinity extends into the geological past as well. Kubota et al.^42^ reconstructed the mid-Holocene (7 Ka BP) to present freshwater budget for the U1429 region, employing Heshang cave δ^18^O as a proxy for the isotopic composition of the freshwater end member. They found that local seawater δ^18^O and river discharge have similar responses (no change in the mean) from 7 Ka to present whereas Heshang δ^18^O indicates a 2.5‰ increase, a trend previously interpreted to represent decreased rainfall^43^. This finding, no systematic reduction in monsoon rainfall from the mid-Holocene to present, is consistent with a range of independent and diverse rainfall proxies from the EAM region^26^ and indicates a decoupling of YRV precipitation amount and precipitation δ^18^O in the past.

Air mass backtrack trajectory analysis indicates both continental and marine moisture sources to the YRV during the modern summer and winter seasons, with 59% of precipitation falling during the summer monsoon months (June through September; Supplementary Fig. 1)^27^. Detailed analysis of YRV moisture sources^44^ indicates that the contribution of moisture from land roughly equals that from the Pacific and Indian Oceans combined during both summer (May–September) and winter (October–April). The relative contributions of modern continental and marine moisture sources over the course of the seasonal cycle, weighted by their isotopic composition, successfully replicates modern cave calcite δ^18^O in the EAM region^27^.

Precipitation δ^18^O (δ^18^O_precip_) values reflect the origin, temperature, humidity and transport history of the water vapor^31^. These factors are significantly different for terrestrial and marine air masses contributing to modern EAM rainfall during summer and winter^40,45^. Analysis of cave drip water^32^ indicates that δ^18^O_precip_ variability in the modern monsoon region of China cannot be explained by either temperature or precipitation alone, likely due to the multiple interacting vapor sources. In addition, Liu et al.^46^ examined the Global and Chinese Network of Isotopes in Precipitation (GNIP and CHNIP) finding that δ^18^O_precip_ in the YRV region is best related to water vapor content of the atmosphere; the amount effect is minor. Hence, there is little evidence, at least from modern data, that EAM water-isotope proxies can be interpreted directly as indicators of local rainfall amount. For these reasons, reconstructing ECS local δ^18^O_sw_ is important, providing an independent proxy indicator of changes in ECS salinity, varying as a function of precipitation and runoff.

### Water-isotope proxy drivers

The oxygen isotopic composition of ECS planktonic foraminifer (δ^18^O_pf_) and YRV δ^18^O_cave_ have a number of drivers in common as well as a number of drivers that influence each independently (Supplementary Fig. 2). Both records reflect global-scale changes in source water δ^18^O as well as local δ^18^O_precip_ since both are under the influence of the EAM, and linked by YRV runoff. Changes in surface temperature, in contrast, may be expressed differently in these two proxies. The dominant impact on δ^18^O_pf_ will be changes in the temperature of calcite precipitation; the impact of evaporation will be minimal because precipitation plus runoff dominates over evaporation in the ECS, where annual average salinity values range between 30 near the coast and 34.5 offshore, out to 130°E^47^. Changes in temperature affect δ^18^O_precip_ and can also be propagated into the cave environment, influencing the temperature of calcite precipitation. Temperature may also have a significant influence through changes in soil zone evaporation and evaporation in the cave atmosphere itself^30,31^. Duan et al.^32^. monitored monthly to bi-monthly δ^18^O_precip_ and cave drip water δ^18^O (δ^18^O_dw_) at 34 sites in the cave region of China to evaluate the impact of processes in the soil, epikarst, and cave environment. They found that 82% of the drip sites showed little variation in δ^18^O_dw_ throughout the 3-year study, indicating that δ^18^O_cave_ incorporates multi-year average signals modulated by seasonally changing recharge and evaporation regimes. Twelve percent of the drip sites recorded damped seasonal to monthly δ^18^O_dw_, compared to δ^18^O_precip_. Six percent fell between the two extremes, with constant, low δ^18^O_dw_ during the wet season and variable, relatively high δ^18^O_dw_ in the dry season, thought to result from flow switching in the karst or evaporation within the cave. James et al.^30^ made the case that seasonal cave ventilation is an important driver of cave-air CO_2_ and hence calcite precipitation; the result being that speleothems from temperate and boreal regions, for example, can be biased toward cool-season calcite precipitation. None of these factors impact δ^18^O_pf_, calcite precipitated by planktonic foraminifers in the offshore surface environment.

Similarly, biases independent of those impacting δ^18^O_cave_ can impact ECS δ^18^O_pf_. Sediment trap results from the Okinawa Trough indicate that *Globigerinoides ruber* specimens in the size fraction used for this study (Methods) are reduced in abundance between December and March, potentially introducing a seasonal bias^48^. Site U1429 is located at the bifurcation point of the Kuroshio Current and Tsushima Warm Current^49,50^; variability in these current regimes could impact δ^18^O_pf_, although the path of the Kuroshio appears stable with regard to glacial sea level changes^51^. Migration of the shoreline closer to Site U1429 during glacial-age sea level low stands (120 m isobath, Supplementary Fig. 3) may alter the delivery and extent of mixing between ^18^O-enriched seawater from the Kuroshio Current with ^18^O-depleted river water at Site U1429. The relative influences of these various independent δ^18^O_pf_ and δ^18^O_cave_ drivers may vary at different time scales.

### Foraminifera δ^18^O

**Fig. 2:**
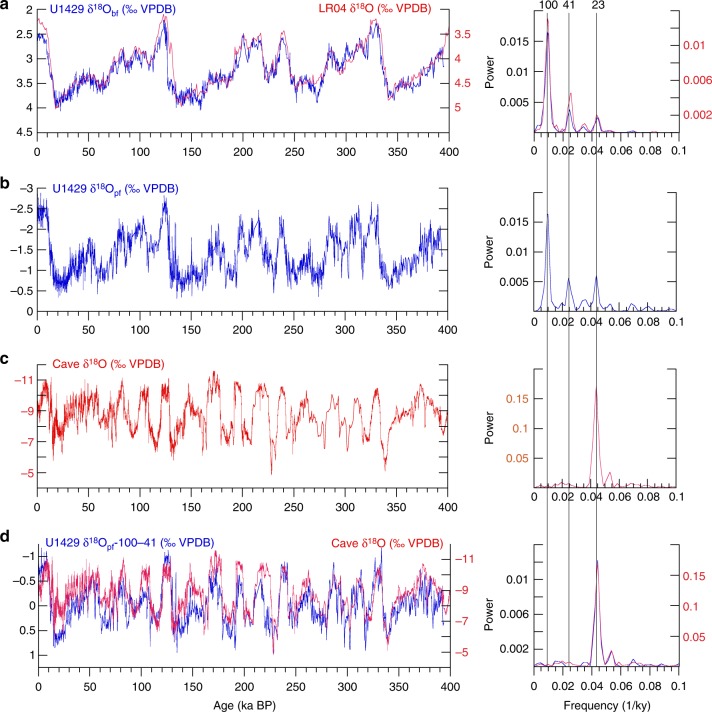
Proxy time series and spectra. **a** U1429 δ^18^O_bf_ mapped to the benthic chronology^52^. **b** U1429 δ^18^O_pf_ on the benthic age model. **c** δ^18^O_cave_^15^, **d** Notched δ^18^O_pf_ mapped to δ^18^O_cave_. Labeled dashed lines denote primary orbital frequencies (frequency = 1/period in ky)

The U1429 benthic foraminifer δ^18^O (δ^18^O_bf_) record was mapped to the global benthic isotope stack^52^ to produce a traditional benthic age model (Methods, Fig. 2a). Cross-spectral analysis indicates high coherence and near-zero phase across the Earth-orbital eccentricity, obliquity, and precession bands, verifying the U1429 chronostratigraphy (Supplementary Fig. 4).

The U1429 δ^18^O_pf_ time series is dominated by eccentricity with lesser, sub-equal variance in the obliquity bands and precession bands (Fig. 2b). In contrast, YRV δ^18^O_cave_^15^ is dominated by precession-band variance (Fig. 2c), a primary reason why the EAM has been interpreted as a system responding dominantly and directly to northern hemisphere summer-insolation forcing^5,14^. The very strong degree to which δ^18^O_cave_ variance is also embedded in offshore δ^18^O_pf_ is illustrated by notch filtering δ^18^O_pf_ (removing eccentricity- and obliquity-band variance) and then mapping the sub-orbital structure to δ^18^O_cave_, creating a cave-based age model (Methods); all further analyses use the cave-based age model. Notched δ^18^O_pf_ (Fig. 2d) demonstrates that the precession-band structure in YRV δ^18^O_cave_ resides in δ^18^O_pf_ as well; the spectra are nearly indistinguishable. This strong orbital-scale correspondence is not due to fine-tuning the benthic age model by correlating the notched δ^18^O_pf_ to δ^18^O_cave_; the cave-based age model is spectrally indistinguishable from the benthic age model (Supplementary Fig. 4). δ^18^O_bf_ on the two age models is highly coherent (0.99 CI) with near-zero phase at the eccentricity (0.08 ± 0.35 ky), obliquity (−0.23 ± 0.29 ky) and precession (0.23 ± 0.17 ky) bands.

**Fig. 3:**
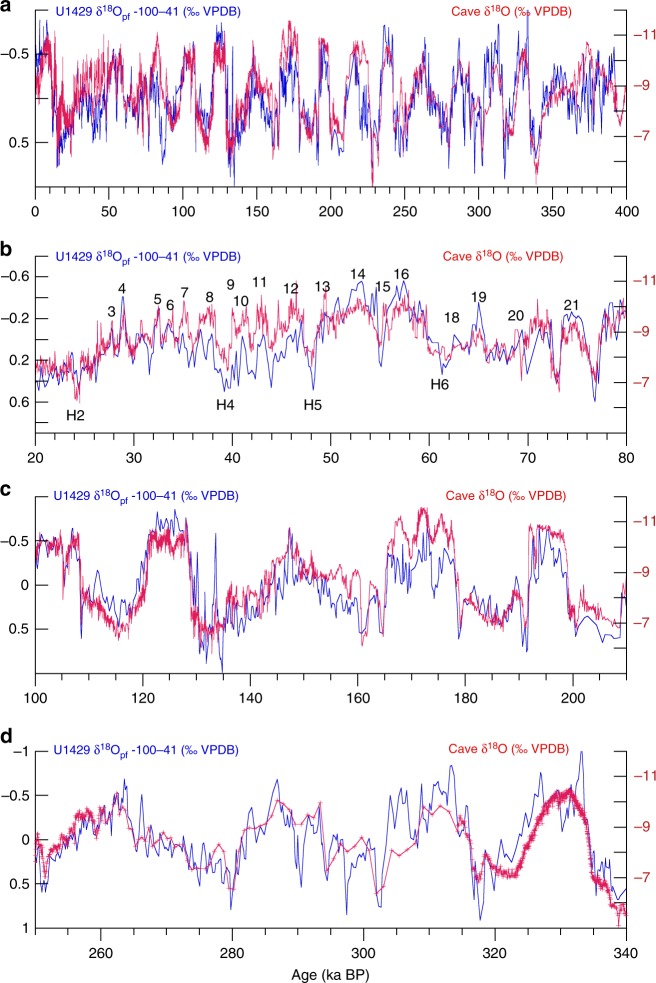
Millennial-scale structure in notched δ^18^O_pf_ compared to δ^18^O_cave_. **a** Notched U1429 δ^18^O_pf_ on the cave-based age model. **b** Expanded 20–80 ka interval with millennial-scale Dansgaard–Oeschger (DO) and Heinrich events numbered. **c** Expanded 100–210 ka interval. **d** Expanded 250–340 ka interval showing structure not previously resolved in δ^18^O_cave_

The strong similarities extend to the sub-orbital scale as well. δ^18^O_pf_ and δ^18^O_cave_ have the same millennial-scale structure during both glacial and interglacial intervals (Fig. 3b, c), including Heinrich and Dansgaard–Oeschger events. Finally, U1429 δ^18^O_pf_ has enhanced resolution of sub-orbital-scale structure within the interval 250 to 340 ka where δ^18^O_cave_ is less resolved or smoothly varying (Fig. 3d). Strong similarities at the orbital (precession) and sub-orbital (millennial) scales confirm that δ^18^O_pf_ and δ^18^O_cave_ share common drivers at these time scales, both likely monitoring the δ^18^O of local precipitation; potential biases that impact each of the two proxies independently are not apparent in these records.

### Seawater δ^18^O

From a climate change standpoint, however, reconstruction of rainfall amount is an important goal. Interpretation of water-isotope proxies as rainfall amount has been a point of contention given that modern data, described above, show that δ^18^O_precip_ and rainfall amount are uncorrelated in the YRV region. This is addressed by quantitatively removing the effects of changing sea surface temperature (SST) and global seawater δ^18^O from δ^18^O_pf_ (Methods, Supplementary Note 1). This yields a record of local δ^18^O_sw_, a proxy that is linearly related to salinity^42,53,54^, responding strongly to YRV runoff as well as direct precipitation to the ECS^41^.

**Fig. 4:**
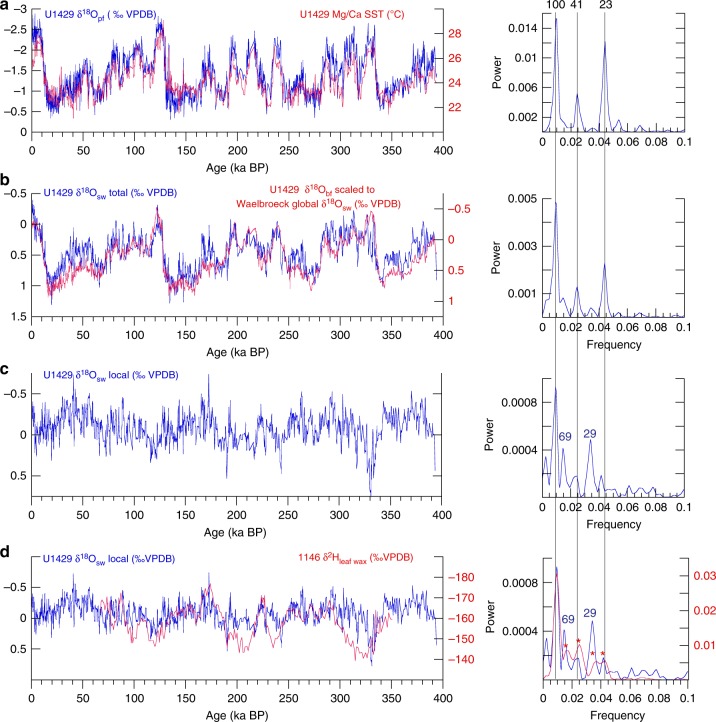
Time series used in deriving local δ^18^O_sw_, and associated spectra. **a** U1429 δ^18^O_pf_ (and spectrum) with Mg/Ca SST, both derived from *G. ruber* (250–355 μm size fraction). **b** U1429 total δ^18^O_sw_ (and spectrum) with U1429 δ^18^O_bf_ scaled to match the global δ^18^O_sw_ curve^74^; this approach preserves the age model and high temporal resolution of the U1429 data. **c**, **d** U1429 local δ^18^O_sw_ (and spectrum) with the Pearl River Valley δ^2^H_wax_ record (and spectrum) from Site 1146^34^. Labeled dashed lines denote primary orbital frequencies (frequency = 1/period in ky). Orbital-scale peaks for frequencies <0.05 labeled with * do not meet the *p* = 0.05 threshold level for probability of chance occurrence (Methods)

**Fig. 5:**
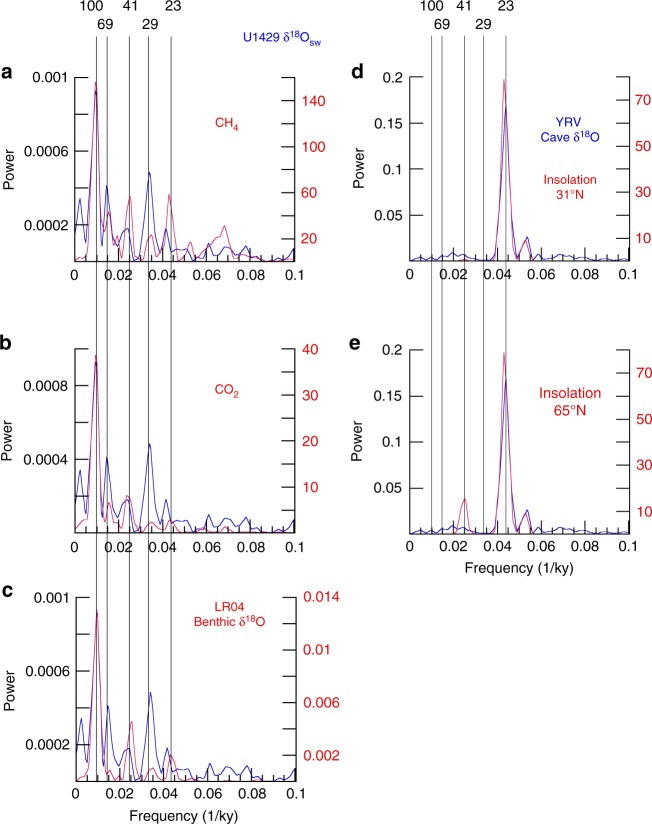
Spectral comparisons of EAM proxy records with global ice volume, greenhouse gases, and insolation. Local δ^18^O_sw_, a record for which the effects of temperature and seawater δ^18^O have been removed, compared to **a** global CH_4_, **b** global CO_2_, and **c** global ice volume. δ^18^O_cave_, a record for which the effects of temperature and seawater δ^18^O have not been removed, compared to **d** local summer and **e** high-latitude summer-insolation forcing. Labeled lines denote primary orbital frequencies (frequency = 1/period in ky)

Removing the SST signal from δ^18^O_pf_ (Fig. 4a) yields the total δ^18^O_sw_ (Fig. 4b). Removing global seawater δ^18^O from total δ^18^O_sw_ (Fig. 4b) yields local δ^18^O_sw_ (Fig. 4c). In contrast to δ^18^O_cave_, local δ^18^O_sw_ has almost no precession-band variance. Instead, it is dominated by variance at primary orbital periods, including 100 ky (eccentricity), 41 ky (obliquity), and both heterodynes thereof (29 and 69 ky). While this spectral structure is very different from that of YRV δ^18^O_cave_, it shares strong similarities with precipitation-isotope records to the north and south as well as with global-scale changes in CO_2_, CH_4_, and terrestrial ice volume (Fig. 5a–c).

The eccentricity-band (100 ky) variance in local δ^18^O_sw_ is coherent (0.92 CI) and near-zero phase (4 ± 2 ky) with the Site 1146 (Pearl River) δ^2^H_wax_ record^34^ located ~1000 km south of the YRV; both are dominated by 100 ky eccentricity-band variance with little to no precession-band variance (Fig. 4d). The Site 1146 record dominantly reflects input from the Pearl River but has contributions from Taiwan and Luzon as well^10,55^. The heterodyne periods (29 and 69 ky) are consistent as well with the 31- and 72-ky heterodynes that dominate variance in the Lantian–Weinan loess δ^2^H_wax_ record^10^ ~300 km north of the YRV.

### Transient model simulations

Seasonality and the extent to which the local δ^18^O_sw_ proxy is consistent with model rainfall simulations is assessed by comparison with results from a 300,000 year long accelerated transient climate simulation using the Community Climate Systems Model version 3.5 (CCSM3)^56^. The CCSM3 simulation (Methods, Supplementary Note 2) was run with varying orbital insolation, greenhouse gasses, ice volume, and sea level, following methods and boundary conditions detailed in Chen et al.^57^, in order to capture the climate response to transient forcing^57,58^. EOF comparison of the model and modern EAM precipitation fields for summer and winter seasons are consistent with one another (Supplementary Fig. 5). These simulation results have been previously used in the EAM region to assess the transient response to changing surface temperature and monsoon precipitation^10,59^.

**Fig. 6:**
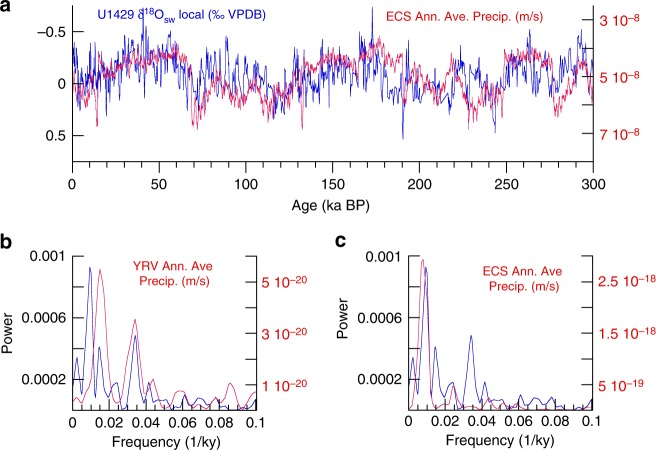
Comparison of model (annual average, red) and U1429 (blue) time series and spectra. **a** U1429 local δ^18^O_sw_ and East China Sea (ECS) model annual average precipitation. The U1429 record is consistent with **b** annual average precipitation over the Yangtze River Valley (YRV) accounting for the 29- and 69-ky variance and **c** ECS annual average precipitation, accounting for the 100- and 41-ky variance. Supplementary Figure 6 shows comparisons of δ^18^O_cave_ (for which temperature has not been removed) with model temperature and precipitation (monthly annual, maximum, and minimum)

Having removed the effects of surface temperature and global seawater δ^18^O from δ^18^O_pf_, the result (local δ^18^O_sw_) is compared to model precipitation. Both the YRV and ECS regions (Fig. 1) were evaluated for monthly maximum (summer), minimum (winter), and annual average precipitation in order to assess spatial and seasonal variability. The local δ^18^O_sw_ heterodyne variance at the 29- and 69-ky periods is unique to the YRV annual average model precipitation field (Fig. 6), suggesting it is transmitted to the ECS via runoff. The 100-ky variance is found in all three (maximum, annual average, and minimum) ECS precipitation records, indicating decreased precipitation during glacial intervals. The same set of proxy-model comparisons was conducted for YRV δ^18^O_cave_, including model temperature since the impact of changing local temperature is unknown. δ^18^O_cave_ is well matched to model YRV surface temperature maximum and precipitation maximum (Supplementary Fig. 6). Hence, the degree to which changes in local temperature or precipitation drive YRV δ^18^O_cave_ cannot be differentiated with these data. More in-depth interpretation of δ^18^O_cave_ and local δ^18^O_sw_ would benefit from isotope-enabled transient model simulations that incorporate realistic greenhouse gas, ice volume, and sea level boundary conditions.

## Discussion

At the eccentricity band, light U1429 δ^18^O_sw_ and Pearl River Valley δ^2^H_wax_ occur during glacial intervals (Fig. 4d). Light glacial-age δ^2^H_wax_ may result from rainout over the emergent continental shelf resulting in lighter inland precipitation isotopes, increased rainout along the moisture transport path (including the emergent Sunda Shelf)^60^, changes in glacial-age source water locations, or cooler local precipitation condensation temperatures. Differentiation among these possible mechanisms, or some combination thereof, is not possible at this time but will benefit from rapidly evolving isotope-enabled simulations that incorporate realistic changes in boundary conditions, including ice-volume and sea level. Interpreted in the context of salinity (rainfall and YRV runoff), the 100-ky variance in the local δ^18^O_sw_ signal is likely influenced by shoreline migration, bringing the YRV freshwater source closer to the site during glacial sea level low stands^61^, counteracting the effect of decreased precipitation indicated by an array of independent proxy records of EAM rainfall^11,26,62^ and model simulations^63–65^.

Heterodyne variance in U1429 and in the Lantian–Weinan section to the north likely reflect interactions of variance associated with eccentricity and obliquity forcing as described in Thomas et al.^10^. Thomas et al. note a significant difference in the spectral structure of East Asian water-isotope proxy records (δ^18^O_cave_ and δ^2^H_wax_) relative to those of loess-based summer monsoon (magnetic susceptibility) and winter monsoon (grain size) proxy records^26^. The loess-based records, also including δ^13^C of inorganic carbonate^11^ and an array of isotopic^12^ and magnetic property proxies^9,26,62^ for summer monsoon precipitation are dominated by primary orbital periodicity (eccentricity, obliquity, and precession). The water-isotope proxies, in contrast, contain large amounts of variance at heterodyne periods, the concentration of which increases from south to north. Thomas et al.^10^ attribute the northward increase in heterodyne variance (spectral complexity) to the increasing influence of multiple environmental parameters contributing to the water-isotope signal; records in the south are primarily dominated by summer monsoon variance with simple spectra, composed mostly of primary orbital-scale variance peaks, whereas water-isotope records further north are influenced by both summer- and winter-monsoon variability (i.e., temperature, winter- and summer-monsoon precipitation) resulting in more complex spectra that include heterodyne variance. Our δ^18^O_sw_ record is consistent with this interpretation, having a combination of primary and heterodyne variance.

The spectral signatures of most EAM proxies are not dominated by 23-ky variance but, rather, by spectral variance at the 100- and 41-ky periods more characteristic of late Pleistocene global ice volume and greenhouse gas forcing^9–12,26,34^. The spectral signature of ECS local δ^18^O_sw_, a record for which the impact of temperature and seawater δ^18^O have been removed, is also well matched to that of CH_4_^66^, CO_2_^67^, and the global benthic δ^18^O stack^52^ (Fig. 5a–c). All are dominated by eccentricity-band variance with lesser amounts of obliquity-band variance. With the exception of CH_4_, all have the least amount of variance in the precession-band. The precession-band variance in CH_4_ (co-equal with obliquity-band variance) has been attributed, in part, to tropical wetland sources^66^. In any case, these records, and their spectral signatures, bear little resemblance to that of direct local or high-latitude summer-insolation forcing (Fig. 5d, e). These findings indicate that the EAM does not respond dominantly and directly to external insolation forcing but, rather, is strongly sensitive to the internal redistribution of this energy, resulting in spectral signatures more similar to those of greenhouse gasses and global ice volume.

In summary, the precession- and millennial-band structure characteristic of YRV δ^18^O_cave_ is also found offshore in ECS δ^18^O_pf_. However, after quantitative removal of the local temperature and global seawater δ^18^O signals, the resulting local δ^18^O_sw_ record (a proxy for rainfall and YRV runoff) no longer matches δ^18^O_cave_. Local δ^18^O_sw_ is dominated by eccentricity- and obliquity-band variance as well as heterodynes thereof, not by precession-band variance as in δ^18^O_cave_. The local δ^18^O_sw_ spectrum, similar to an array of other proxies across the EAM region, is consistent with that of CO_2_, CH_4_, and global ice volume. This indicates that EAM rainfall variability is more sensitive to internal forcing mechanisms related to high-latitude ice sheet and greenhouse gas variability, and less sensitive to direct insolation forcing, as is commonly inferred on the basis of the precession-dominated YRV δ^18^O_cave_ spectrum.

## Methods

### Planktonic oxygen and carbon stable isotopes

U1429 was sampled at 5 cm resolution from 0 to 25 m below sea floor (mbsf), at 5 or 10 cm resolution from 25 to 35 mbsf, and at 5, 10, or 15 cm resolution from 35 to 181 mbsf. Samples were freeze-dried, wet-sieved at 63 μm, and dried in an oven at 50 °C. The >63 μm fraction was then sieved into four size fractions, 63–150, 150–250, 250–355, and >355 μm. Approximately 50 individual *G. ruber* (white, sensu stricto) were picked from the 250–355 μm size fraction for both stable isotope (δ^18^O, δ^13^C) and Mg/Ca analysis. Approximately 30 and 20 individuals were used for Mg/Ca and stable isotope analyses respectively. Samples for stable isotope analyses were sonified in ethanol to remove fine clays, homogenized, and subsampled (~80 μg CaCO_3_) for analysis on the Brown University MAT252 IRMS coupled to a Kiel III carbonate device. Samples were reacted by individual acid addition (99% H_3_PO_4_ at 70 °C). A total of 2031 *G. ruber* samples and a total of 314 standards (BYM and Carrara) were analyzed. Repeated analyses of Brown Yule Marble (*n* = 116, 1σ) yields −2.27 ± 0.03 for δ^13^C and −6.48 ± 0.07 for δ^18^O. Carrara Marble (*n* = 198, 1*σ*) yield 2.03 ± 0.03 for δ^13^C and −1.89 ± 0.05 for δ^18^O. Replicate analysis of homogenized foraminifera samples (*n* = 42, 1*σ*) yields ± 0.05 for δ^18^O and ± 0.03 for δ^13^C. All results were calibrated to National Institute of Standards and Technology (Gaithersburg, Maryland) carbonate isotope standard NBS 19 and are reported as ‰ VPDB.

### Benthic oxygen and carbon stable isotopes

U1429 was sampled at 10, 15, 25, or 30 cm. Samples were dried in an oven at 40 °C, wet-sieved at 63 μm, and dried in an oven at 40 °C. Three to ten well-preserved tests (*Uvigerina* spp, *C. wuellerstorfi* depending on availability) were broken into large fragments, cleaned in alcohol in an ultrasonic bath, then dried at 40 °C. In a few samples, where foraminiferal density was low, only 1–2 specimens were analyzed. Measurements were made with the Finnigan MAT 251 mass spectrometer at the Leibniz Laboratory, Kiel University. The instrument is coupled on-line to a Carbo-Kiel Device (Type I). Samples were reacted by individual acid addition (99% H_3_PO_4_ at 73 °C). Standard external error is better than ±0.07‰ and ±0.05% for δ^18^O and δ^13^C, respectively. Replicate measurements on ~5% of samples indicate mean reproducibility better than ±0.11‰ and ±0.13‰ for δ^18^O and δ^13^C, respectively. Results were calibrated using the National Institute of Standards and Technology (Gaithersburg, Maryland) carbonate isotope standard NBS 19 along with three internal standards, and are reported on the PeeDee belemnite (VPDB) scale.

### SST and δ^18^O_sw_ reconstruction

Mg/Ca analysis of *G. ruber* sensu stricto (ss) was carried out for U1429 at intervals of approximately 10, 15, 25, or 30 cm, using splits of the same *G. ruber* fraction used to measure planktonic δ^18^O. Prior to measurement, foraminiferal tests were cleaned following the reductive approach, modified from Boyle and Keigwin^68^, as detailed in refs.^41^ and ^42^. The metal/Ca ratios of most of samples were determined with a Thermo Scientific ELEMENT XR, a double focusing sector field inductively coupled plasma mass spectrometer (ICP–MS) at the Mutsu Institute for Oceanography (MIO); 87 samples in MIS 5 were determined with an ELEMENT 2 at the University of Toyama^41^. The elements ^24^Mg and ^44^Ca, measured in a middle resolution mode, were used to determine Mg/Ca for the samples analyzed at the MIO. The precision of the measurement was checked by replicate measurement (every five to six samples) of working standards produced at MIO from high purity standards (1000 ± 3 mg/L) SPEX Claritas PPT. Relative standard deviation (RSD) of the working standards was <2.6% at MIO. The CaCO_3_ reference material CRM was used as a working standard and RSD of its replicate analyses was 1.1% at the University of Toyama. The accuracy of Mg/Ca ratios were confirmed by analyses of a CaCO_3_ reference standard, coral *Porites* standard material JCp-1, whose Mg/Ca values was internationally determined (4.199 ± 0.065 mmol/mol^69^,). The measured Mg/Ca of JCp-1 was 4.161 ± 0.064 (1σ, *N* = 24) mmol/mol at MIO and 4.148 ± 0.055 (1σ, *N* = 11) mmol/mol at the University of Toyama. Based on the difference of Mg/Ca between two laboratories, 0.0131 mmol/mol was added for the Mg/Ca data measured at the University of Toyama. In addition to Mg/Ca, Mn/Ca were measured to monitor the contamination by diagenetic coating. Mn/Ca of 99% of the samples were less than 0.5 mmol/mol. As there was no positive relationship between Mg/Ca and Mn/Ca (>0.5 mmol/mol), we did not eliminate high Mn/Ca samples.

Sixteen samples were repicked and rerun for duplication test for Mg/Ca in MIO. The average of the difference of Mg/Ca between duplicates was 0.086 ± 0.149 (1σ) mmol/mol, which was equivalent to 0.48 ± 0.36 °C. The effect of preferential removal of Mg^2**+**^ from foraminiferal calcite on Mg/Ca values due to dissolution on the sea floor (e.g., ref.^70^ and references therein) is likely negligible because the water depth of the core site (732 m) is well above the modern lysocline (~1600 m) in the ECS and over 800 m above the depth at which dissolution impacts the Mg/Ca temperature estimation in this region^41^.

Seawater δ^18^O is derived using Paleo-Seawater Uncertainty Solver (PSU Solver^71^) No regional Mg/Ca calibration exists for the East China Sea. We employ the Mg/Ca calibration of Tierney et al.^72^ that utilizes all available culture data in a multivariate calibration that accounts for both salinity and temperature. This calibration has an exponential slope of 8.4 ± 1.5%/°C, consistent with that previously used in the East China Sea^41^ which has an exponential slope of 8.9%/°C but does not account for salinity. We utilize the seawater δ^18^O-temperature relationship (low light) of Bemis et al.^73^, the ECS *G. ruber* core top seawater δ^18^O-salinity relationship of Horikawa et al.^41^, and the global sea level curve of Waelbroeck et al.^74^. Instead of using the Waelbroeck curve directly, the U1429 benthic δ^18^O record was scaled to match the Waelbroeck curve such that the age model and high-resolution sampling at U1429 remains intact. Scaling is accomplished by normalizing the U1429 benthic δ^18^O record followed by multiplication of each sample by 0.313 (the standard deviation of the Waelbroeck curve) and addition of 0.364 to align the core top to a value of 0.27‰. Propagated uncertainty (1σ) in δ^18^O_sw_ is assessed using the following error estimates on the underlying parameters (δ ^18^O_pf_ ± 0.1‰, Mg/Ca ± 0.2 mmol/mol, and age ± 2 ky). The resulting time series and spectra are shown in Supplementary Figure 8a.

We assess sensitivity to the temperature term and calibration approach by comparing our Seawater δ^18^O result to that derived using SST from Eq. (3) of Gray et al.^75^. Equation (3) from Grey et al. expresses Mg/Ca as a function of temperature, salinity, and pH using calibration data from 440 globally distributed plankton tow and sediment trap samples. The temperature sensitivity (6 ± 0.8%/°C) is considerably smaller than that in the Tierney equation (8.4 ± 1.5%/°C). Sediment trap and tow data use natural, open ocean conditions relative to laboratory-controlled conditions. pH is derived using a simple linear regression of *G. ruber* δ^18^O vs. pH from the ODP Site 999A data presented in Fig 9a of Foster et al.^76^:

1 $${\mathrm{pH}} = 0.045^\ast \,\delta^{18}{\mathrm{O}} + 8.25\,\left( {n = 29,\,r^2 = 0.76,\,p > 0.0001} \right)$$

Equation 1, applied to U1429 *G. ruber* δ^18^O, yields a first-order estimate of surface water pH for the past 400 kyrs, presuming the calibration from 999A (Caribbean Sea) is appropriate for U1429 (East China Sea). PSU solver is used to derive local seawater δ^18^O for comparison with results from the Tierney 2015 calibration equation. The result is shown in Supplementary Fig. 8b; despite the differences in the temperature coefficients and underlying calibration approaches, both results are dominated by 100-kyr variability with very little precession-scale variance.

Quantitative reconstruction of salinity is not attempted, given the unconstrained nature of the δ^18^O_sw_ salinity relationship over time^23,54,77^. No reliable information is available on the potentially variable slope of the δ^18^O_sw_-salinity relationship in the ECS over the past 400,000 years. Such evaluation awaits reliable, time-dependent isotope-enabled simulations with realistic boundary conditions^23^.

SST was also reconstructed using the U^K’^_37_ approach. Bulk sediment samples (3 g) were taken from the core at 10 cm intervals for alkenone analysis. Long-chain alkenones were extracted from freeze-dried sediment samples. Organic compounds were extracted using an accelerated solvent extractor (ASE 200, Dionex) with a solvent mixture (CH_2_Cl_2_:CH_3_OH, 99:1 v/v) at high temperature (100 °C) and pressure (1500 psi). The extracts were cleaned by elution (3 × 500 μl CH_2_Cl_2_) through a silica cartridge. Saponification was performed at 80 °C for 2 h with 300 μl of 0.1 M KOH in 90/10 CH_3_OH/H_2_O. The neutral fraction, containing the alkenones, was obtained by partitioning into hexane. After being concentrated under N_2_, the final extract was analyzed using a gas chromatograph (Agilent 7890 A) equipped with a flame ionization detector and a DB-1 column (60 m × 0.32 mm i.d.). Temperatures were calculated using the alkenone unsaturation index (U^K’^_37_) and the calibration equation of Prahl et al.^78^ (U^K’^_37_ = 0.034 T + 0.039; Supplementary Fig. 7). Reproducibility of alkenone temperatures for replicate samples of a homogeneous marine sediment lab standard run during the project is better than ±0.2 °C (*n* = 124, 2σ). Duplicate analyses from U1429 is ± 0.4 °C (*n* = 31 2σ). Supplementary Fig. 8c shows local seawater δ^18^O reconstructed using the U1429 U^K’^_37_ SST record instead of the U1429 Mg/Ca record; the result is again a record dominated by 100-kyr variance with very little 23-kyr variance, as expected on the basis of Supplemental Fig. 7 which shows the same precession-band variance is as in Mg/Ca-derived SST, but greater 100-kyr variance.

### U1429 age model

A traditional marine chronostratigraphy (benthic age model) was established by mapping the U1429 benthic δ^18^O to the global benthic stack^52^ over the past 400 ky (Supplementary Table 1). This was accomplished by correlation of structure in the two records using the Linage function in Analyseries^79^. Cross-spectral analysis documents high coherence (>0.95CI) and near-zero phase at the eccentricity (−2 ± 0.7 ky), obliquity (0.5 ± 0.5 ky) and precession bands (−0.7 ± 0.3 ky) (Supplementary Fig. 4). The 100- and 41-ky variance was then removed from planktonic δ^18^O (benthic age model) by notch filtering the 100- and 41-ky variance (Methods). The result was then mapped to the Cheng et al.^15^ composite cave δ^18^O record using the Analyseries Linage function (Supplementary Table 1). This fine-tuning of the U1429 benthic age model (cave-based age model) is justified on the basis that benthic δ^18^O on the two age models are highly coherent (>0.99 CI) with near-zero phase at the eccentricity (0.08 ± 0.35 ky), obliquity (−0.23 ± 0.29 ky) and precession (0.23 ± 0.17 ky) bands.

### CCSM3 simulations

Paleoclimate simulations were performed using the National Center for Atmospheric Research (NCAR) Community Climate System Model (CCSM) version 3.5^56,80^ in the fully coupled configuration (active atmosphere, ocean, land, and sea ice). The ocean model has a zonal resolution that varies from 340 km at the equator to 40 km around Greenland and 350 km in the Northern Pacific. This spatially varying resolution is achieved by placing the north pole of the grid over Greenland and reflects the different relevant length scales of the two processes that are important in maintaining a stable global climate; deep convection around Greenland and in the Arctic as well as ocean heat uptake at the equator. In the vertical there are 25 depth levels; the uppermost layer has a thickness of 8 m and the deepest layer has a thickness of 500 m. This and the atmospheric, land, and sea-ice models operate under the T31_3_3 setup^80^, which have been developed specifically for long paleoclimate and biogeochemistry applications.

Insolation was calculated using orbital parameters from Berger and Loutre^13^. Atmospheric CO_2_ and CH_4_ were prescribed (CO_2_, IGBP PAGES/WDCA contribution series number: 2008–055^81^; CH_4_, IGBP PAGES/WDCA contribution series number: 2008–054^66^.) Continental ice sheets and sea level (i.e., land-ocean boundaries) were prescribed by scaling the ICE-5G ice distribution for the LGM to present^82^ to the marine benthic δ^18^O record^52^and saving these parameters as boundary conditions for the model. At the end of each year of the simulation, the orbital parameters and the atmospheric greenhouse gases were advanced by 100 years. When the model boundary conditions changed (e.g., land-ocean boundaries changing due to sea level rise or fall equivalent to 40 m), all the components of the model were reconfigured manually and the model was restarted using the previous year as the initial conditions. This acceleration technique, similar to those used in other paleoclimatic modeling studies^2,83–85^, enables us to gain insight into the temporal evolution of the climate system given restricted computer resources. Several studies have shown that acceleration factors of 10 and 100 produced similar results^84^. Jackson and Broccoli^83^, for example, used an acceleration factor of 30 in a simulation of the past 165,000 years. The upper ocean and the atmosphere reach quasi-equilibrium in this acceleration approach^2^. In our acceleration approach, the change in insolation forcing is small (<0.5 W/m) from year to year at all latitudes in the shortest orbital cycle (precession)^58^.

The experiment consisted of three separate simulations, beginning with an insolation only simulation, followed by addition of greenhouse gasses, followed by addition of ice-volume and sea level (Fox-Kemper et al., manuscript in preparation). Successively adding boundary conditions is a useful method for understanding the sensitivity of the model response to these forcing mechanisms. Results in this manuscript are from the simulation using the full set of boundary conditions. Model precipitation data were averaged for the regions depicted in Fig. 1. Monthly maximum, minimum, and annual average model output were used in comparison with proxy data. Use of monthly maximum and minimum values (as opposed JJA, DFJ) accounts for the fact that the timing of maximum insolation, in the northern hemisphere (for example), can occur in May, June, or July depending on the orbital configuration and is thus, not tied to any single orbital configuration (e.g., June 21 Perihelion).

Currently, accelerated models assume that Earth’s orbit is fixed, meaning that changes in the length and strength of Earth’s seasons due to changes in Earth’s orbital parameters are not accounted for in the definition of the calendar upon which monthly values are computed. While this is not a major issue for Holocene-scale studies^84,86^, it becomes important for orbital-scale simulations^2,57,58,83^. Therefore, it is important to identify and correct for this calendar effect if one wishes to accurately relate paleoclimate model results to real-world paleoclimate records. The calendar problem was corrected for (Nelson A.D. et al., manuscript in preparation) by following the methodology of other works^57,87,88^.

### Time series analysis

Cross-spectral analyses were performed with the Blackman–Tukey approach using Analyseries software^79^. All spectra employed a Bartlett window and a 30% (*n*/3) lag where n is the number of series data points. For model-proxy comparisons, the bandwidth is 0.0167, non-zero coherence is >0.3844, and the error estimation on the power spectrum is 0.6255. For proxy–proxy comparisons, the bandwidth is 0.0127, non-zero coherence is >0.3844, and the error estimation on the power spectrum is 0.6255.

Notching (removing) the 100-kyr (eccentricity) and 41-kyr (obliquity) variance was accomplished by filtering (Gaussian) at a central frequency of 0.01 and a bandwidth of 0.005 for eccentricity and a central frequency of 0.0245 and a bandwidth of 0.0035 for obliquity.

Individual linear spectra were calculated using the Analyseries periodogram function and a Bartlett window. The periodogram produces an unsmoothed spectrum (equivalent to the Blackman–Tukey with a 100% lag). It is useful in that it does not smooth away side lobes that may be present due to, for example, time scale inaccuracies. Since Analyseries does not have a means of assessing confidence, the Periodogram tool on the NASA Exoplanet Archive site (http://exoplanetarchive.ipac.caltech.edu/cgi-bin/Pgram/nph-pgram) was used to assess orbital-scale spectral peaks discussed in the manuscript for frequencies < 0.05 (periods greater than 20 ky) using the Lomb-Scargle algorithm^89^. Peaks discussed in the manuscript at frequencies < 0.05 labeled with * do not meet the *p* = 0.05 threshold level for probability of chance occurrence.

## Electronic supplementary material

Supplementary Information

## Data Availability

All data necessary to assess the validity of this research are presented in the paper and Supplementary Materials. The paleoclimate proxy data are archived at NOAA’s National Centers for Environmental Information (NCEI).
