# Supplementary material for: Precession-band variance missing from East Asian monsoon runoff
Source: Nat Commun. 2018 Aug 22;9:3364. doi: 10.1038/s41467-018-05814-0 (PMC6105601; doi:10.1038/s41467-018-05814-0)
Supplement: Supplementary file 1 — Supplementary Information [file 41467_2018_5814_MOESM1_ESM.pdf]

Supplementary Information

Precession-band variance missing from East Asian monsoon runoff

S. C. Clemens, A. Holbourn, Y. Kubota, K.E. Lee,
Z. Liu, G. Chen, A. Nelson, B. Fox-Kemper

Supplementary Note 1. Sea Surface Temperature Records

Averaged over the entire record, Mg/Ca SST is 23.9±1.6°C (1σ), 2.1 °C warmer than $U^{K'}_{37}$ (21.8±1.6°C; 1σ) (Supplementary Fig. 7). This is consistent with a core-top offset of 2.2°C measured for these proxies at nearby cores[1]. The largest differences are found during glacial intervals (MIS 6, 8, and 10) where $U^{K'}_{37}$ SST can approach 4°C cooler. This is reflected in the spectral character of the two records at the eccentricity band. However, both proxies have the same amounts of precession- and obliquity-band variance, lending confidence to our seawater $\delta^{18}O$ reconstruction at these orbital bands. Paired Mg/Ca-$\delta^{18}O$, both measured on the same *G. ruber* fraction[2-4], are used here to reconstruct $\delta^{18}O_{sw}$ (Methods).

Supplementary Note 2. CCSM3 Model Results in the East Asian Monsoon Region

Southeast China is characterized by a strong meridional (tripole) gradient in summer monsoon precipitation (Supplementary Fig. 5). The tripole structure is characterized by high precipitation within the Yangtze River Valley (YRV) and lower precipitation to the north and south[5-7]. As summarized by Hsu et al.[5], the tripole pattern has been linked to atmospheric wave structure(s) associated with anomalous heating over the Philippine Sea, anomalous heating over the Tibetan Plateau, and Rossby wave perturbations forced by anomalous heating over the Eurasian continent. These multiple associations lead to the hypothesis that the tripole results from amplification of an intrinsic dynamic mode that can be triggered by a number of different mechanisms. Regardless of the mechanism, EOF analysis of summer and winter model precipitation demonstrates spatial patterns similar to those found in the modern EAM region, comparing well with the Global Climatology Precipitation Project (GPCP) version 2.3 data[8,9] (Supplementary Fig. 5) and with the modern transient runs of the Community Earth System Model (CESM) Large Ensemble Community Project (LENS)[10] as well (Fox-Kemper, B. et al. PHASEMAP Part I: Orbital, greenhouse, and glacial impacts on 300ka of climate variability in an accelerated forcing earth system model. Paleoceanography (in prep). The primary (first) EOF of EAM summer and winter precipitation in the paleoclimate simulations do not have modern analogues (Supplementary Fig. 5); these EOFs arise from precipitation variability at orbital timescales, much longer than those in current modern observations, and would not be captured in modern observational data.

Supplementary Table 1. Age model tie points.

Benthic Age Model		Cave-based Age Model	
Depth (m, core composite depth below Sea Floor (CCSF-D)	Age (ka BP; Lisiecki and Raymo 2005, Benthic $\delta^{18}O$)	Depth (m, CCSF-D)	Age (ka BP; Cheng et al., 2016, Cave $\delta^{18}O$)
0.82	2.07	0.22	0.49
3.07	8.94	3.82	9.64
8.65	18.09	5.43	12.36
21.84	37.94	8.65	16.11
27.85	45.00	18.10	29.14
34.55	55.00	18.54	30.79
43.29	75.05	19.11	32.48
46.75	84.06	19.54	33.73
49.90	89.88	20.24	35.09
53.13	90.04	20.84	36.05
56.75	96.13	22.03	38.39
59.19	102.84	22.83	39.53
60.81	108.82	23.28	40.04
64.56	122.90	23.85	41.23
70.69	134.97	24.40	42.62
90.60	166.93	25.44	44.01
103.20	199.48	27.32	46.46
106.52	210.16	28.98	48.23
110.29	216.89	30.62	49.39
119.40	237.05	31.81	53.04
120.86	242.43	33.21	55.11
125.80	242.58	34.48	58.76
130.30	251.96	35.71	59.93
145.25	283.40	37.80	65.08
153.06	300.41	42.04	72.22
160.72	329.06	43.00	73.33
165.29	341.07	43.86	76.05
181.14	393.27	48.71	88.51
		50.09	90.56
		53.17	91.81
		54.09	94.39
		57.31	100.41
		58.30	105.14
		60.15	108.20
		60.76	108.70
		62.12	115.62
		63.17	117.52
		64.08	120.38
		64.46	121.06
		66.64	128.48
		67.97	130.59
		70.73	133.48

		72.21	136.15
		80.28	147.38
		84.73	157.00
		87.49	160.85
		88.81	164.69
		90.35	165.49
		92.26	169.82
		96.17	175.82
		96.85	178.53
		97.53	179.64
		101.03	191.15
		101.98	193.02
		104.42	200.55
		105.17	208.77
		116.88	229.17
		117.19	232.15
		118.68	234.53
		120.23	242.07
		126.73	243.65
		127.81	249.07
		129.06	250.39
		130.80	251.63
		137.11	263.85
		142.53	280.30
		144.66	282.56
		145.67	286.89
		148.00	289.91
		149.79	294.54
		153.80	302.32
		157.44	317.31
		158.53	323.13
		162.28	334.62
		165.76	338.68
		173.79	366.04
		177.11	377.81

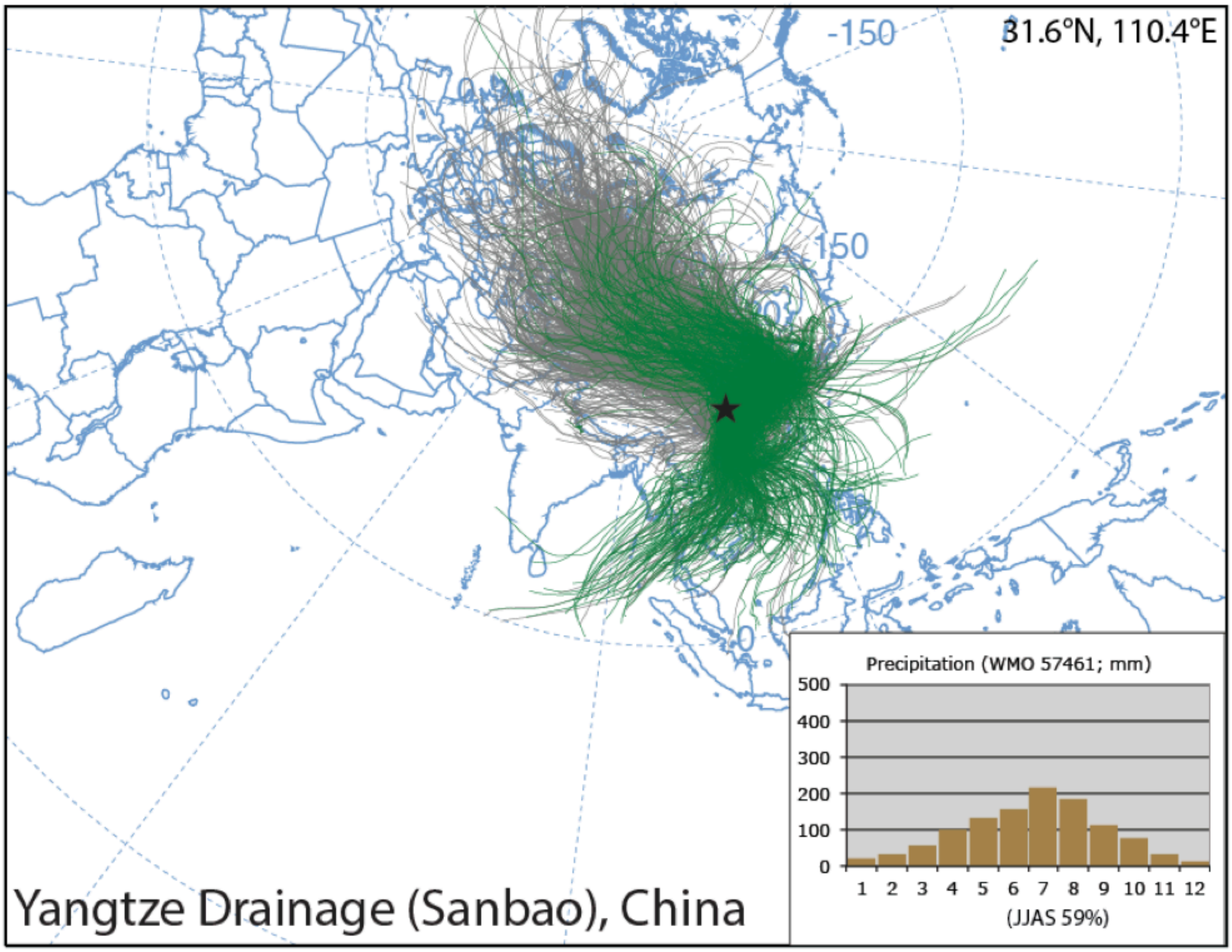

Supplementary Fig. 1. Ten day HYSPLIT backtrack and maps [11,12] of air masses entering the Yangtze River Basin (2007 to 2011). Green trajectories indicate rain-bearing air masses while grey trajectories yielded no rain. Both winter winds from the continental interior and summer winds off the ocean (Bay of Bengal and South China Sea) carry precipitation to the Yangtze River Valley. Inset: long-term monthly World Meteorology Organization (Site #57461) rainfall climatology.

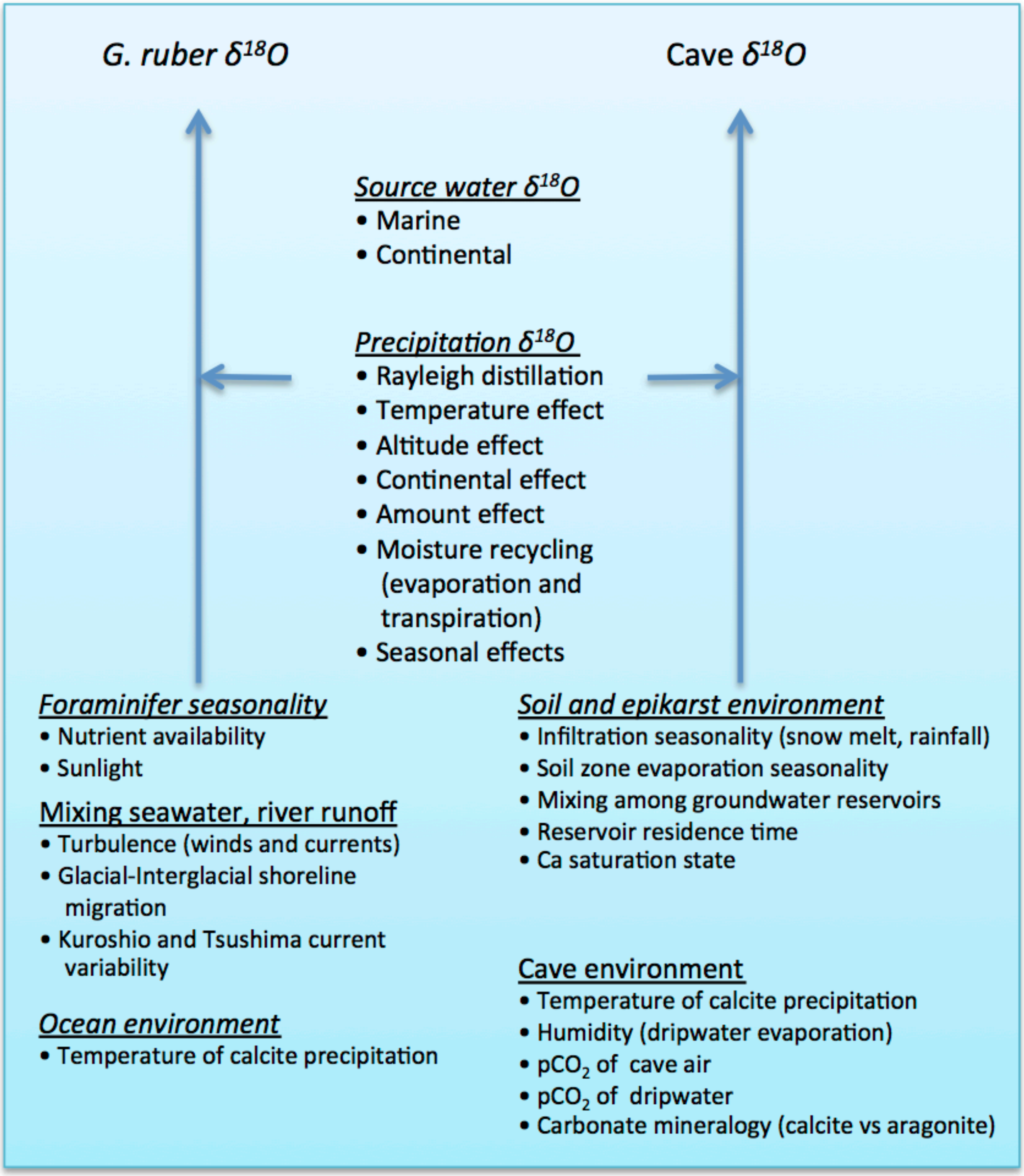

Supplementary Fig 2. Comparison of factors with potential to influence speleothem and planktonic foraminifer $\delta^{18}O$. Both proxies share variance imparted by changes in source water $\delta^{18}O$ and precipitation $\delta^{18}O$ because they are linked by Yangtze River runoff and East Asian monsoon rainfall. Other factors contribute variance independently to either the onshore (speleothem) or offshore planktonic foraminifer (*G. ruber*) $\delta^{18}O$ proxies.

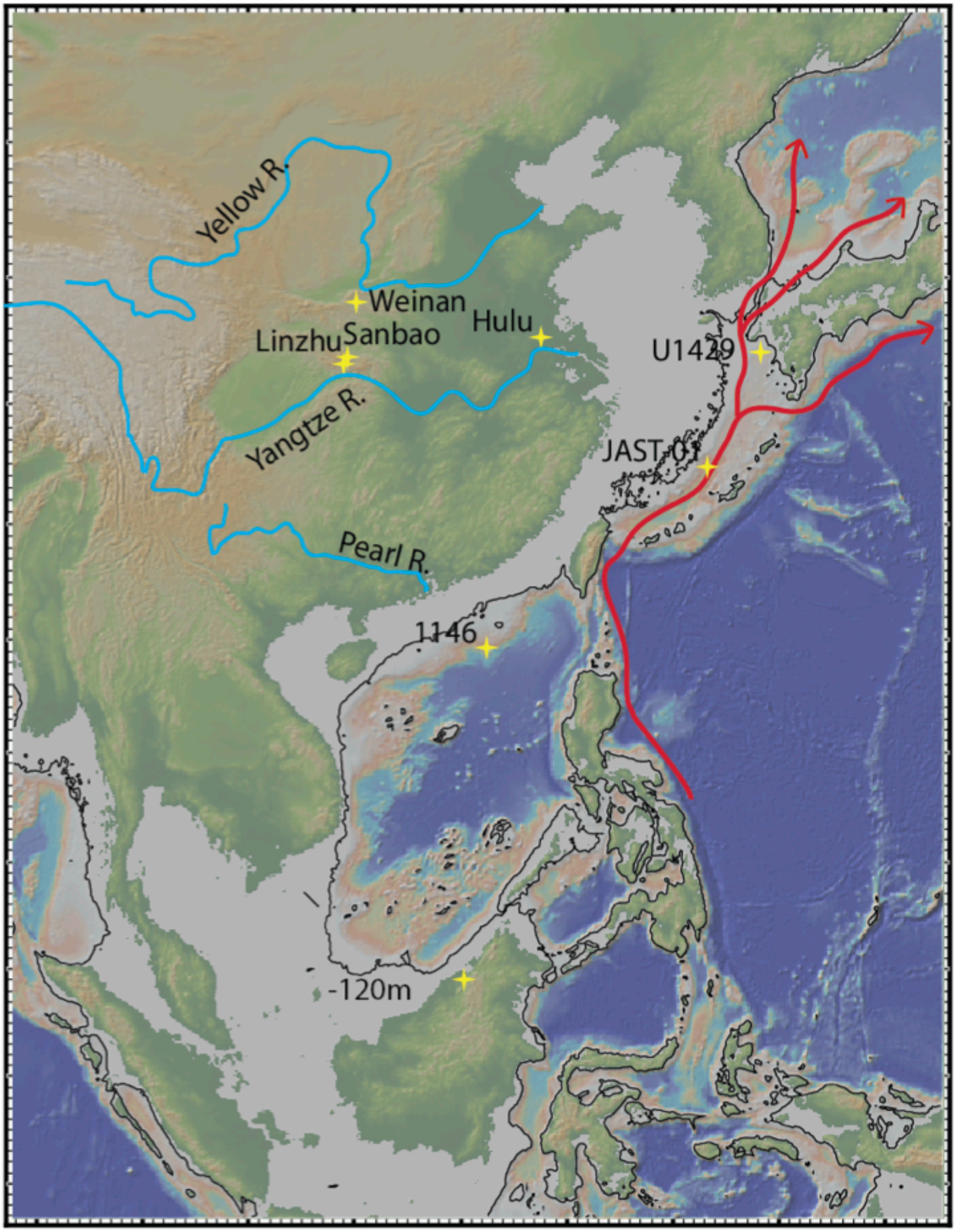

Supplementary Fig. 3. Location map showing site locations, surface currents (red) and 120 m isobath (black). Map created using GeoMapApp (http://www.geomapapp.org) using the Global Multi-Resolution Topography data set[13].

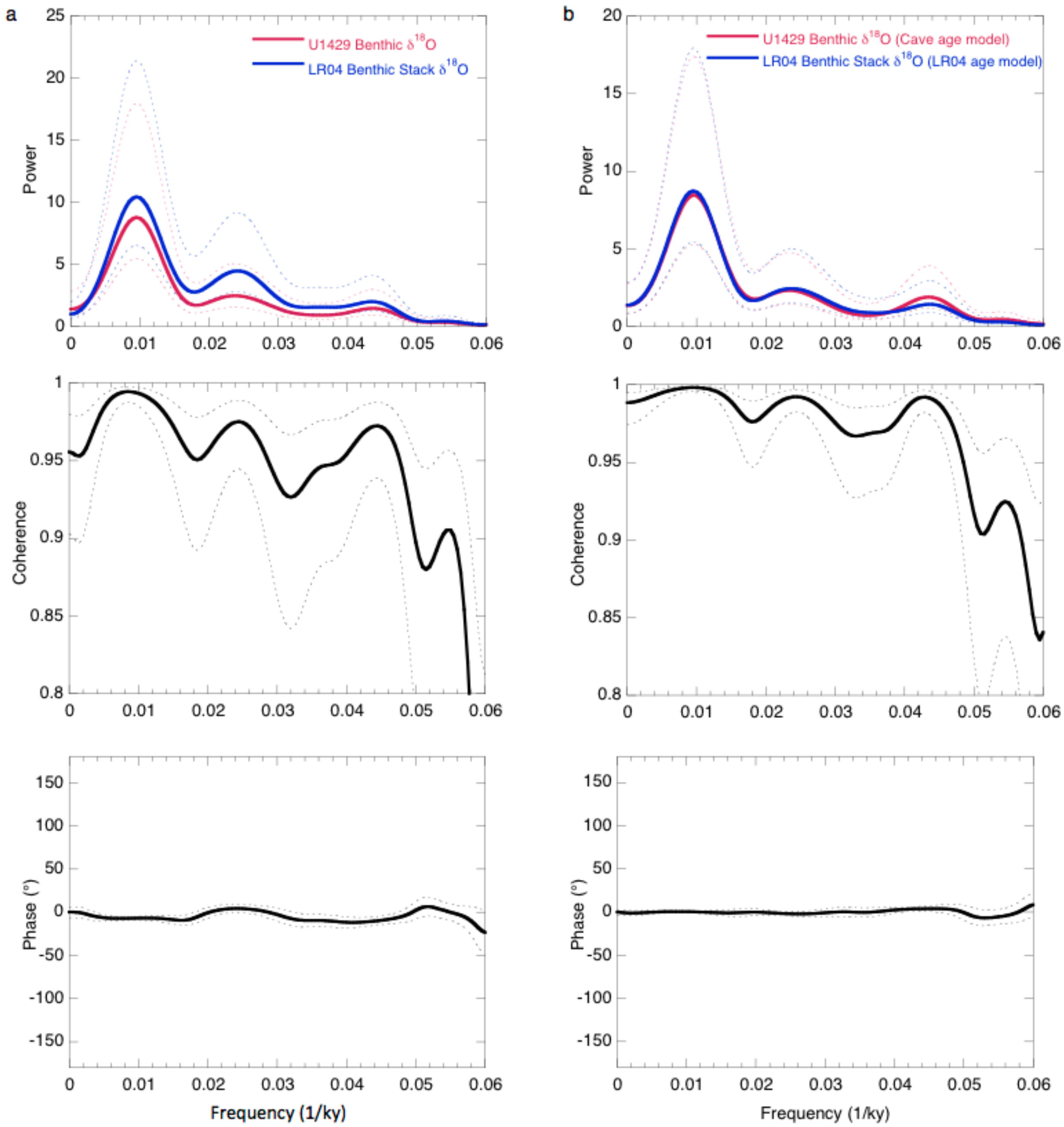

Supplementary Fig. 4. Cross-spectral coherence and phase comparisons. (a) U1429 $\delta^{18}O_{bf}$ on the benthic age model compared to LR04 global stacked $\delta^{18}O$[14]. (b) U1429 $\delta^{18}O_{bf}$ on the benthic age model compared to U1429 $\delta^{18}O_{bf}$ on the cave-based age model.

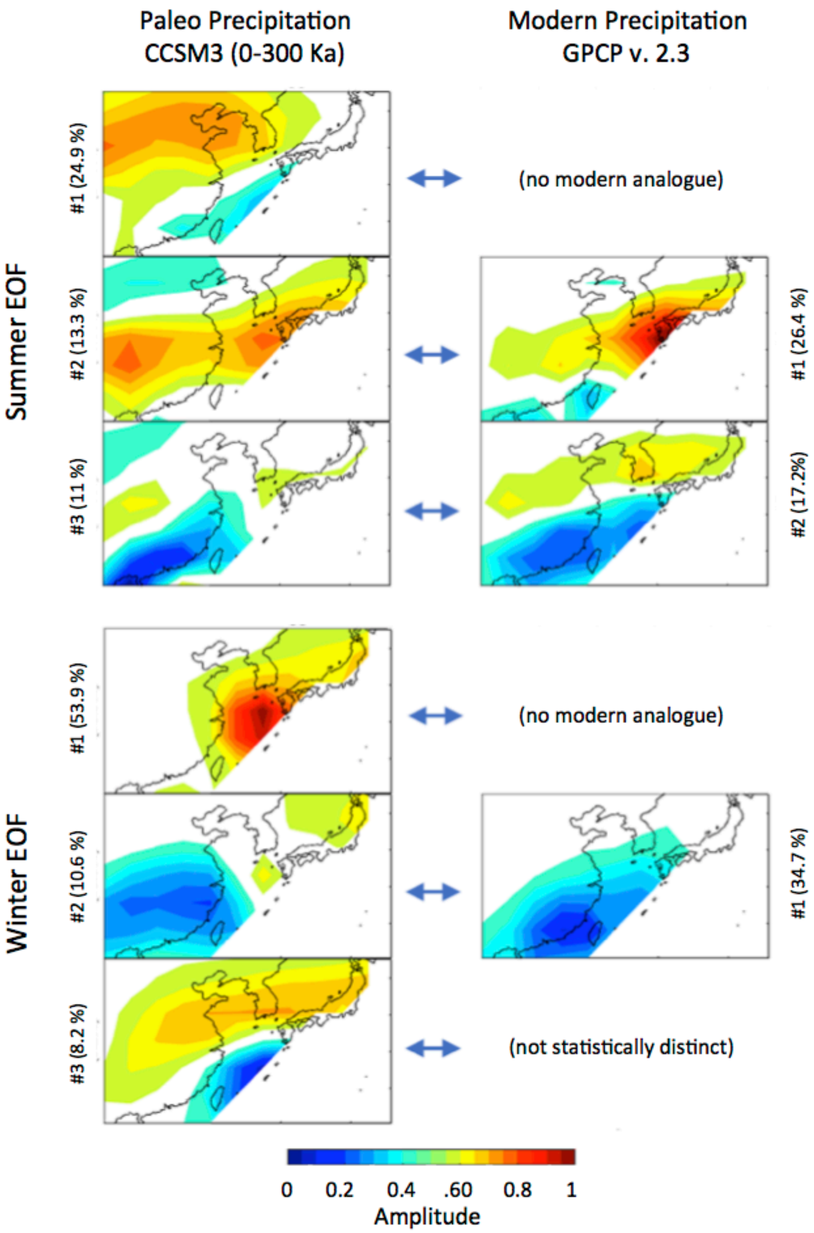

Supplementary Fig. 5. EOF comparison of CCSM3[15] climate simulation precipitation (monthly mean, 300 ka to present, 100x acceleration; Fox-Kemper, B. et al. PHASEMAP Part I: Orbital, greenhouse, and glacial impacts on 300ka of climate variability in an accelerated forcing earth system model. Paleoceanography, in prep.) to GPCP V.2.3 combined satellite and gauge monthly precipitation data (1979-2016)[8,9]. Model summer (April-Sept.) precipitation EOFs 2 and 3 are consistent with GPCP EOFs 1 and 2. Model winter (Oct. – March) EOF 2 is consistent with GPCP EOF 2. Principle component time series of model EOF 1 (summer) has strong 23-kyr variability, possibly associated with insolation-forced changes in the latitudinal position of the northern hemisphere Jet[16] and would not be captured in modern data. PC time series of model EOF 1 (winter) has strong 100-kyr variability, likely associated with glacial-interglacial exposure and flooding of the continental shelf and would not be captured in modern data.

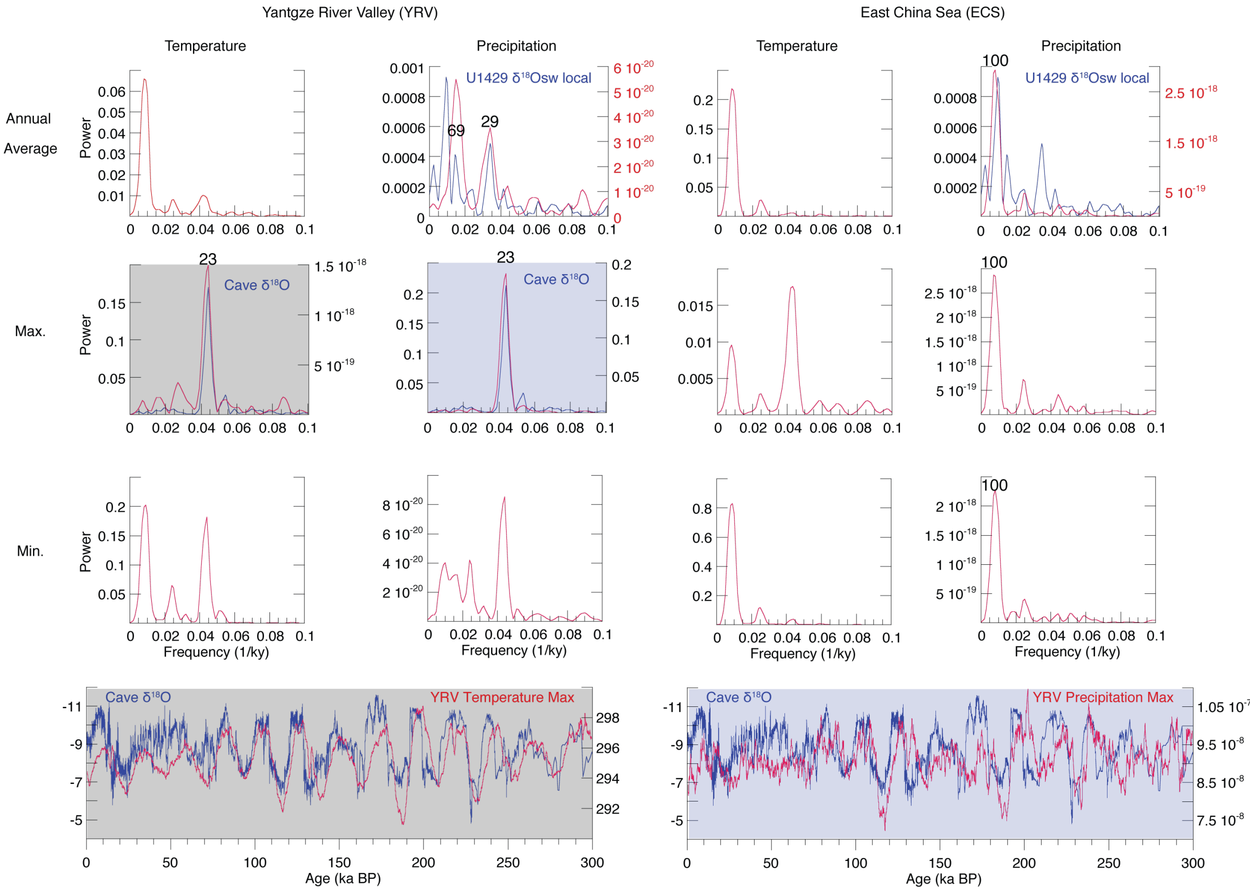

Supplementary Fig. 6. Model spectra (red) for annual average, maximum, and minimum temperature (K) and precipitation (ms^{-1}) in the Yangtze River Valley and East China Sea. The U1429 local $\delta^{18}O_{sw}$ spectrum (blue), for which temperature has been removed, is consistent with annual average precipitation in the Yangtze River Valley and East China Sea. The $\delta^{18}O_{cave}$ spectrum (blue), for which temperature has not been removed, is consistent with maximum monthly temperature and precipitation in the Yangtze River Valley; time series of these model parameters are plotted with $\delta^{18}O_{cave}$ (bottom panel). Spectral peak significance is as described in manuscript Fig. 2 caption.

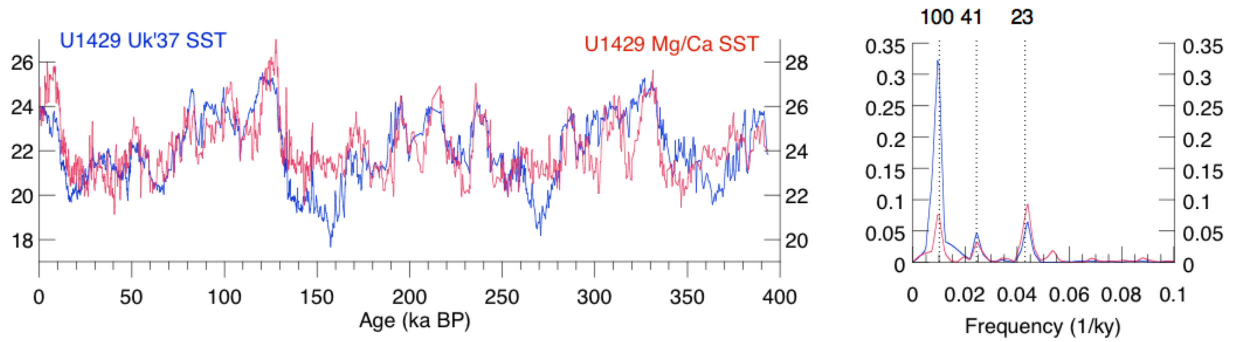

Supplementary Fig. 7. Mg/Ca and $U^{k'}_{37}$ SST reconstructions at U1429. Both records have the same amounts of variance at the obliquity (41-ky) and precession (23-ky) bands while $U^{k'}_{37}$ SST has greater eccentricity-band (100-ky) variance, driven by relatively cooler glacial age temperatures during MIS6 (~130 to 160 Ka) and MIS8 (~265 to 280 ka). Spectral peak significance is as described in manuscript Fig. 2 caption.

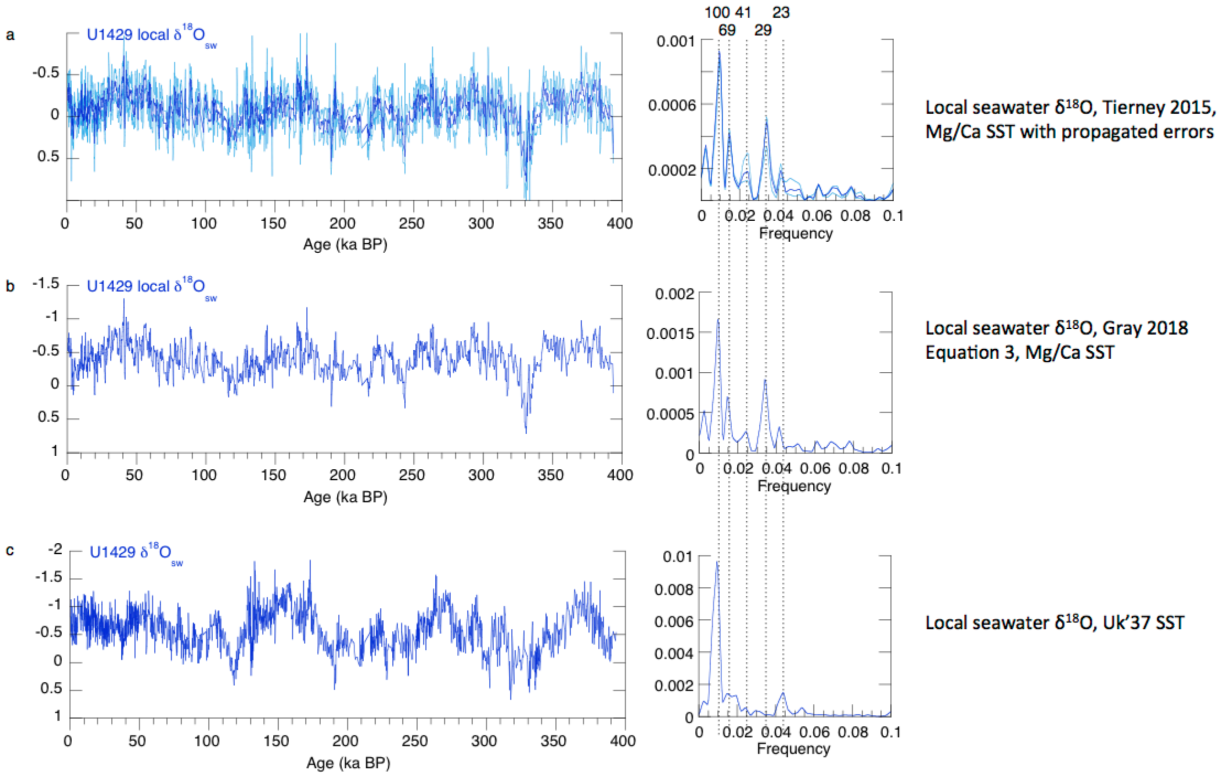

Supplementary Fig. 8. Local $\delta^{18}O_{sw}$ reconstructions comparing use of Mg/Ca SST relationships of Tierney et al.,[17] (including ±1σ propagated errors shown in light blue; Methods) (a) with that of Gray et al.,[18] (b), and with that using $U^{K'}_{37}$ SST instead of Mg/Ca SST (Methods) (c). The result that precession-scale variance is removed from the spectrum when temperature and global ice-volume are accounted for is robust across differences in the temperature coefficient used (0.06 to 0.084), the calibration methodology (laboratory culture versus plankton tow and sediment trap data), and SST proxy (Mg/Ca- and $U^{k'}_{37}$ –based reconstructions).

Supplementary References

1 Kim, R. A., Lee, K. E. & Bae, S. W. Sea surface temperature proxies (alkenones, foraminiferal Mg/Ca, and planktonic foraminiferal assemblage) and their implications in the Okinawa Trough. *Progress in Earth and Planetary Science* **2**, 1-16, doi:10.1186/s40645-015-0074-1 (2015).

2 Elderfield, H. & Ganssen, G. Past temperature and delta O-18 of surface ocean waters inferred from foraminiferal Mg/Ca ratios. *Nature* **405**, 442-445, doi:10.1038/35013033 (2000).

3 Lea, D. W., Pak, D. K. & Spero, H. J. Climate impact of late Quaternary equatorial Pacific sea surface temperature variations. *Science* **289**, 1719-1724 (2000).

4 Nürnberg, D., Muller, A. & Schneider, R. R. Paleo-sea surface temperature calculations in the equatorial east Atlantic from Mg/Ca ratios in planktic foraminifera: A comparison to sea surface temperature estimates from U-37(K '), oxygen isotopes, and foraminiferal transfer function. *Paleoceanography* **15**, 124-134 (2000).

5 Hsu, H.-H. & Lin, S.-M. Asymmetry of the Tripole Rainfall Pattern during the East Asian Summer. *Journal of Climate* **20**, 4443-4458, doi:10.1175/JCLI4246.1 (2007).

6 Hsu, H.-H. & Liu, X. Relationship between the Tibetan Plateau heating and East Asian summer monsoon rainfall. *Geophysical Research Letters* **30**, doi:10.1029/2003GL017909 (2003).

7 Weng, H. Multi-scale summer rainfall variability over China and its long-term link to global sea surface temperature variability. *Journal of the Meteorological Society of Japan* **77**, 845 (1999).

8 Adler, R. F. *et al*. The Version-2 Global Precipitation Climatology Project (GPCP) Monthly Precipitation Analysis (1979–Present). *Journal of Hydrometeorology* **4**, 1147-1167, doi:10.1175/1525-7541(2003)004<1147:TVGPCP>2.0.CO;2 (2003).

9 Huffman, G. J., Adler, R. F., Bolvin, D. T. & Gu, G. Improving the global precipitation record: GPCP Version 2.1. *Geophysical Research Letters* **36**, n/a-n/a, doi:10.1029/2009GL040000 (2009).

10 Kay, J. E. *et al*. The Community Earth System Model (CESM) Large Ensemble Project: A Community Resource for Studying Climate Change in the Presence of Internal Climate Variability. *Bulletin of the American Meteorological Society* **96**, 1333-1349, doi:10.1175/BAMS-D-13-00255.1 (2014).

11 Stein, A. F. *et al*. NOAA's HYSPLIT Atmospheric Transport and Dispersion Modeling System. *Bulletin of the American Meteorological Society* **96**, 2059-2077, doi:10.1175/BAMS-D-14-00110.1 (2015).

12 Rolph, G., Stein, A. & Stunder, B. Real-time Environmental Applications and Display sYstem: READY. *Environmental Modelling & Software* **95**, 210-228, doi:https://doi.org/10.1016/j.envsoft.2017.06.025 (2017).

13 Ryan, W. B. F. *et al*. Global Multi-Resolution Topography synthesis. *Geochemistry, Geophysics, Geosystems* **10**, Q03014, doi:10.1029/2008GC002332 (2009).

14 Lisiecki, L. E. & Raymo, M. E. A Pliocene-Pleistocene stack of 57 globally distributed benthic δ18O records. *Paleoceanography* **20**, doi:10.1029/2004PA001071 (2005).

15 Shields, C. A. *et al*. The Low-Resolution CCSM4. *Journal of Climate* **25**, 3993-4014, doi:10.1175/JCLI-D-11-00260.1 (2012).

16 Chiang, J. C. H. *et al*. Role of seasonal transitions and westerly jets in East Asian paleoclimate. *Quaternary Science Reviews* **108**, 111-129, doi:https://doi.org/10.1016/j.quascirev.2014.11.009 (2015).
17 Tierney, J. E., Pausata, F. S. R. & deMenocal, P. Deglacial Indian monsoon failure and North Atlantic stadials linked by Indian Ocean surface cooling. *Nature Geoscience* **9**, 46, doi:10.1038/ngeo2603
https://www.nature.com/articles/ngeo2603 - supplementary-information (2015).
18 Gray, W. R. *et al*. The effects of temperature, salinity, and the carbonate system on Mg/Ca in Globigerinoides ruber (white): A global sediment trap calibration. *Earth and Planetary Science Letters* **482**, 607-620, doi:https://doi.org/10.1016/j.epsl.2017.11.026 (2018).
